# Supplementary material for: Diagnostic value of serum procalcitonin, lactate, and high-sensitivity C-reactive protein for predicting bacteremia in adult patients in the emergency department
Source: PeerJ. 2017 Nov 27;5:e4094. doi: 10.7717/peerj.4094 (PMC5708183; doi:10.7717/peerj.4094)
Supplement: Table S1 — Results of the examination of the ability of procalcitonin (PCT) elevation (≥0.5 ng/ml) to continue to predict positive blood culture and blood culture positive for gram-negative bacteria (GNB) or gram-positive bacteria (GPB) in an expanded cohort of similar patients. [file peerj-05-4094-s002.docx]

Supplementary Table 1. Results of the examination of the ability of procalcitonin (PCT) elevation (≥0.5 ng/ml) to continue to predict positive blood culture and blood culture positive for gram-negative bacteria (GNB) or gram-positive bacteria (GPB) in an expanded cohort of similar patients.

| **Cohort* (no. of patients)** | **Elevation of PCT** | **Sensitivity (95% Confidence Interval, CI)** | **Specificity (95% CI)** | **Accuracy (95% CI)** | **Positive-test likelihood (95% CI)** | **Negative-test likelihood (95% CI)** | **Odds Ratio (95% CI)** | ***P* value** |
| --- | --- | --- | --- | --- | --- | --- | --- | --- |
| Initial (n = 886) | To predict pos. BC (n = 527) | 0.81  (0.74–0.86) | 0.47  (0.43–0.50) | 0.54  (0.51–0.57) | 1.51  (1.36–1.66) | 0.41  (0.31–0.55) | 3.64  (2.46–5.51) | <0.0001 |
| Expanded (n = 2,234) | To predict pos. BC (n = 968) | 0.76  (0.71–0.80) | 0.63  (0.60–0.65) | 0.65  (0.63–0.67) | 2.04  (1.87–2.21) | 0.38  (0.31–0.46) | 5.34  (4.08–7.04) | <0.0001 |
|  | | | | | | | | |
| Initial (n = 886) | To predict GNB (n = 479) | 0.88  (0.81–0.93) | 0.47  (0.43 –0.50) | 0.53  (0.50–0.56) | 1.65  (1.49 –0.81) | 0.26  (0.16 – 0.40) | 6.44  (3.65 – 12.15) | <0.0001 |
| Expanded (n = 2,234) | To predict GNB (n = 879) | 0.86  (0.80–0.90) | 0.63  (0.60–0.65) | 0.65  (0.63–0.67) | 2.3  (2.11–2.48) | 0.23  (0.16–0.31) | 10.13  (6.72–15.75) | <0.0001 |
|  | | | | | | | | |
| Initial (n = 886) | To predict GPB (n = 418) | 0.68  (0.57–0.79) | 0.47  (0.43–0.50) | 0.49  (0.45–0.52) | 1.28  (1.06–1.49) | 0.68  (0.47–0.93) | 1.89  (1.11–3.33) | 0.02 |
| Expanded (n = 2,234) | To predict GPB (n = 795) | 0.63  (0.54–0.71) | 0.63  (0.60–0.65) | 0.63  (0.61–0.65) | 1.68  (1.44–1.91) | 0.59  (0.47–0.73) | 2.83  (1.97–4.09) | <0.0001 |

*Two study cohorts: the first is the original research cohort (n = 886) and another is the expanded cohort consisting of all cases who had simultaneous PCT test and blood cultures results (n = 2,234).
