# Supplementary material for: Diagnostic value of serum procalcitonin, lactate, and high-sensitivity C-reactive protein for predicting bacteremia in adult patients in the emergency department
Source: PeerJ. 2017 Nov 27;5:e4094. doi: 10.7717/peerj.4094 (PMC5708183; doi:10.7717/peerj.4094)
Supplement: Table S2 — Results of the examination of the ability of procalcitonin (PCT) elevation (≥0.5 ng/ml) and lactate (LAC) elevation (≥19.8 mg/dl) to continue to predict positive blood culture and blood culture positive for gram-negative bacteria (GNB) or gram-positive bacteria (GPB) in an expanded cohort of similar patients. [file peerj-05-4094-s003.docx]

Supplementary Table 2. Results of the examination of the ability of procalcitonin (PCT) elevation (≥0.5 ng/ml) and lactate (LAC) elevation (≥19.8 mg/dl) to continue to predict positive blood culture and blood culture positive for gram-negative bacteria (GNB) or gram-positive bacteria (GPB) in an expanded cohort of similar patients.

| **Cohort* (no. of patients)** | **Elevation of PCT and lactate** | **Sensitivity (95% Confidence Interval, CI)** | **Specificity (95% CI)** | **Accuracy (95% CI)** | **Positive-test likelihood (95% CI)** | **Negative-test likelihood (95% CI)** | **Odds Ratio (95% CI)** | ***P* value** |
| --- | --- | --- | --- | --- | --- | --- | --- | --- |
| Initial (n = 886) | To predict pos. BC (n = 255) | 0.53  (0.46–0.6) | 0.78  (0.75–0.81) | 0.72  (0.7–0.75) | 2.41  (1.98–2.91) | 0.60  (0.51–0.7) | 3.98  (2.81–5.63) | <0.0001 |
| Expanded (n = 923) | To predict pos. BC (n = 267) | 0.53  (0.46–0.6) | 0.78  (0.75–0.81) | 0.72  (0.69–0.75) | 2.39  (1.97–2.87) | 0.6  (0.51–0.7) | 3.94  (2.81–5.55) | <0.0001 |
|  | | | | | | | | |
| Initial (n = 886) | To predict GNB (n = 224) | 0.58  (0.49 – 0.67) | 0.78  (0.75 – 0.81) | 0.75  (0.72–0.78) | 2.64  (2.14 – 3.22) | 0.54  (0.43 – 0.65) | 4.90  (3.24 – 7.45) | <0.0001 |
| Expanded (n = 923) | To predict GNB (n = 236) | 0.59  (0.5–0.68) | 0.78  (0.75–0.81) | 0.75  (0.72–0.78) | 2.65  (2.16–3.22) | 0.53  (0.42–0.64) | 5.02  (3.33–7.6) | <0.0001 |
|  | | | | | | | | |
| Initial (n = 886) | To predict GPB (n = 184) | 0.45  (0.34–0.57) | 0.78  (0.75–0.81) | 0.75  (0.72–0.78) | 2.06  (1.52–2.70) | 0.7  (0.55–0.85) | 2.93  (1.73–4.96) | <0.0001 |
| Expanded (n = 923) | To predict GPB (n = 193) | 0.44  (0.33–0.56) | 0.78  (0.75–0.81) | 0.75  (0.72–0.78) | 1.98  (1.46–2.6) | 0.72  (0.57–0.86) | 2.75  (1.63–4.61) | <0.0001 |

**Two study cohorts: the first is the original research cohort (n = 886) and another is the expanded cohort consisting of all cases who had simultaneous PCT and lactate testing and blood cultures results (n = 923).
