# Supplementary material for: Diagnostic value of serum procalcitonin, lactate, and high-sensitivity C-reactive protein for predicting bacteremia in adult patients in the emergency department
Source: PeerJ. 2017 Nov 27;5:e4094. doi: 10.7717/peerj.4094 (PMC5708183; doi:10.7717/peerj.4094)
Supplement: Table S3 — Please note that studies cited in the table used different cut-off values of PCT. [file peerj-05-4094-s004.docx]

Supplementary Table 3. Analysis of studies reporting procalcitonin (PCT) as a single decision tool and diagnostic odds ratio (DOR) as a single indicator of bloodstream infection. Please note that studies cited in the table used different cut-off values of PCT.

| Authors | Oussalah et al. | Juutilainen et al. | Lin et al. (this study) | Oussalah et al. | Lin et al. (this study) | Juutilainen et al. | Lin et al. (this study) |
| --- | --- | --- | --- | --- | --- | --- | --- |
| Diagnostic test | PCT ≥ 10 ng/mL | PCT | PCT > 0.5 ng/mL | PCT ≥ 10 ng/mL | PCT > 0.5 ng/mL | PCT | PCT > 0.5 ng/mL |
| Patient number | 1,067 | <65 | 479 | 975 | 418 | <65 | 527 |
| Clinical setting | Diverse | Hematologic patients with neutropenic fever | Adult Emergency Department | Diverse | Adult Emergency Department | Hematologic patients with neutropenic fever | Adult Emergency Department |
| Applicable to Emergency Department (Yes/No) | maybe | Yes | Yes | maybe | Yes | Yes | Yes |
| Type of bacteremia prediction | Gram-negative bacteria | Gram-negative bacteria | Gram-negative bacteria | Gram-positive bacteria | Gram-positive bacteria | Bacteremia, not otherwise specified. | Bacteremia, not otherwise specified. |
| Diagnostic odds ratio (DOR) | 5.98 | 4.14 | 6.44 | 3.64 | 1.89 | 2.63 | 3.64 |
| 95% Confidence Interval of DOR | 5.20–6.88 | 2.00–8.58 | 3.65–12.15 | 3.11–4.26 | 1.11–3.33 | 1.56–4.44 | 2.46–5.51 |
| Reference | (Oussalah et al. 2015) | (Juutilainen et al. 2011) | This study | (Oussalah et al. 2015) | This study | (Juutilainen et al. 2011) | This study |
